# An Unusual Clinical Presentation of Solitary Fibrous Tumor in the Oral Cavity

**DOI:** 10.1155/2017/4395049

**Published:** 2017-02-23

**Authors:** Everton Freitas de Morais, Deborah Gondim Lambert Moreira, Viviane Alves De Oliveira, Rodrigo Rodrigues Rodrigues, Adriano Rocha Germano, Roseana de Almeida Freitas

**Affiliations:** ^1^Oral Pathology Postgraduate Program, Federal University of Rio Grande do Norte, Natal, RN, Brazil; ^2^Division of Oral and Maxillofacial Surgery, Federal University of Rio Grande do Norte, Natal, RN, Brazil

## Abstract

Solitary fibrous tumor is a rare neoplasm of mesenchymal origin that usually affects the pleura. This rarity becomes more relevant in the oral cavity since the clinical features are nonspecific. A 66-year-old female patient presented with a 3-month history of a swelling in the floor of the mouth, measuring 2 cm in greatest diameter, and pain symptomatology. Occlusal and panoramic radiographs showed no bone involvement. Ultrasonography of the submandibular and parotid salivary glands revealed normal morphology, dimensions, and echogenicity. During this exam, a nodular image of low echogenicity measuring about 2.7 × 1.8 cm was detected. An excisional biopsy was performed and histopathological analysis revealed a well-defined tumor-like lesion with alternation between hypercellular areas without a defined pattern and hypocellular areas. On immunohistochemistry, the tumor was positive for CD34 and CD99 and negative for *α*-SMA, S-100, and bcl-2. Combining the histopathological and immunohistochemical features, the diagnosis was solitary fibrous tumor. The patient is under periodical clinical follow-up and shows no signs of recurrence 7 months after surgical excision of the tumor. The combination of clinical-pathological and immunohistochemical features is necessary for the diagnosis.

## 1. Introduction

Solitary fibrous tumor (SFT) is a rare neoplasm of mesenchymal origin that usually affects the pleura [1]. Involvement of unusual sites such as the oral and maxillofacial region has been reported in the literature [2–5].

SFT in the oral cavity is rare and shows no specific clinical characteristics for establishment of the diagnosis [3]. Oral SFT preferably affects the buccal mucosa and tongue of female patients in the sixth decade of life. Clinically, it is a slow-growing, painless well-defined submucosal mass of variable size [4–6]. Histopathological analysis combined with immunohistochemistry is necessary for diagnostic conclusion [4].

Surgical excision is the treatment of choice, but follow-up of the patient is recommended because of the incomplete knowledge of tumor behavior due to its rarity. In addition, reports of possible recurrence of SFT render the behavior of this tumor doubtful and even aggressive in some cases [5]. The use of adjuvant therapies such as radiotherapy and chemotherapy has been reported in cases of incomplete surgical resection or of malignant tumors [6, 7].

Approximately 90 cases of SFT of the oral cavity have been reported in the English language literature. To date, only five cases of SFT have been previously reported in the floor of the mouth [4, 8–11]. This study reports a case of SFT in the floor of the mouth, an uncommon site, discussing the clinical-pathological and immunohistochemical features used for its diagnosis and comparing the findings with recent literature data.

## 2. Case Report

**Figure 1:**
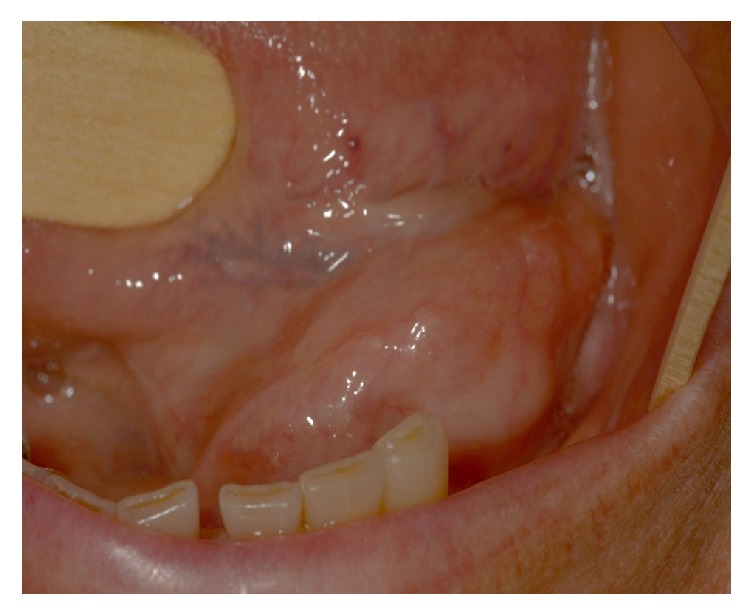
Initial clinical presentation of the patient. Swelling in the floor of the mouth.

A 66-year-old white female patient was referred to the Oral and Maxillofacial Surgery Service of the Federal University of Rio Grande do Norte with a 3-month history of a swelling in the floor of the mouth and pain symptomatology. Intraoral physical examination showed a mucosa colored swelling of hard consistency in the left sublingual region that measured approximately 3 cm in its greatest diameter (Figure 1).

**Figure 2:**
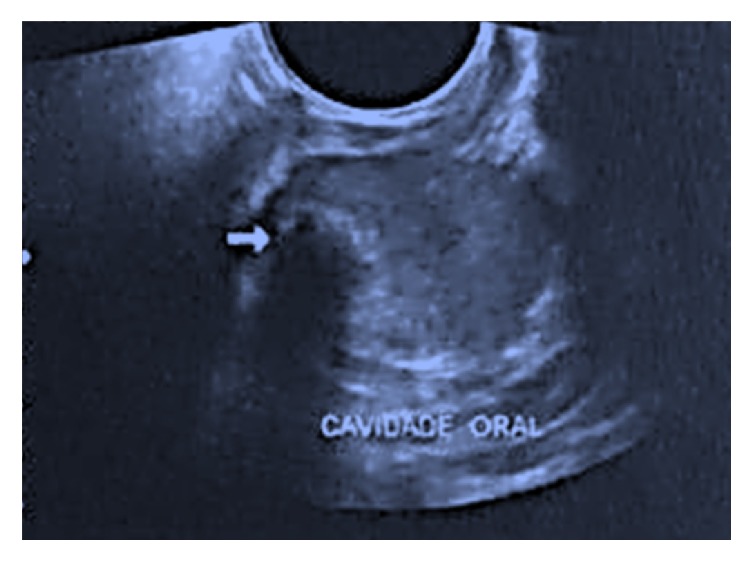
Ultrasonography of the salivary glands. A nodular image of low echogenicity measuring about 2.7 × 1.8 cm was detected.

No facial alterations or palpable lymph nodes were detected upon extraoral examination. Occlusal and panoramic radiographs showed that the lesion only affected the soft tissues and no bone involvement was observed. Ultrasonography of the submandibular and parotid salivary glands revealed normal morphology, dimensions, and echogenicity. During this exam, a nodular image of low echogenicity measuring about 2.7 × 1.8 cm was detected (Figure 2). The nodule contained a hyperechogenic focus of 0.9 cm situated in the topography of the left sublingual region that caused posterior acoustic shadowing. The clinical diagnosis was pleomorphic adenoma.

**Figure 3:**
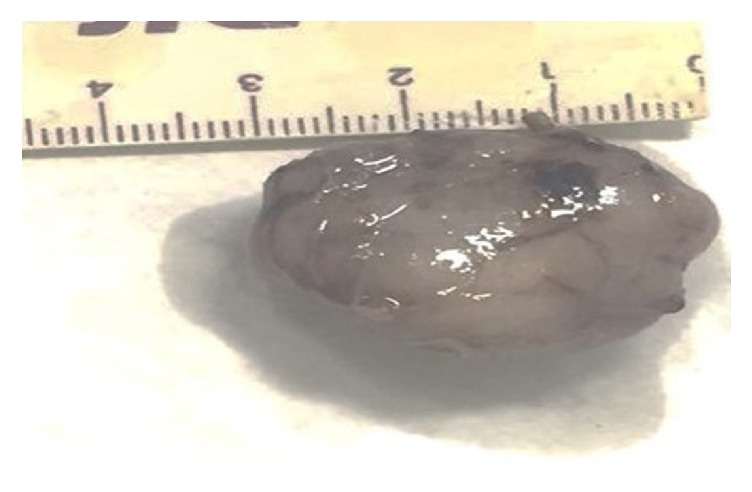
Surgical specimen. Surgical specimen measuring 3 cm in its greatest diameter upon macroscopic inspection.

An excisional biopsy was performed and analysis of the surgical specimen revealed an encapsulated, oval lesion with a smooth surface and brown color (Figure 3). Histopathological analysis showed a well-defined tumor-like lesion with alternation between hypercellular areas without a defined pattern and hypocellular areas.

**Figure 4:**
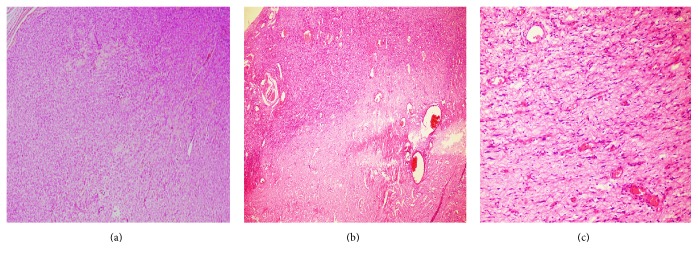
Histopathological features (hematoxylin-eosin). (a) Fragment of the mesenchymal neoplasm showing the proliferation of spindle-shaped cells. The tumor appeared as a well-circumscribed mass with a fibrous capsule. (b) Rich vascularization with hypocellular and hypercellular areas. (c) Enlarged view of spindle-shaped cells exhibiting mild pleomorphism in focal areas.

The neoplastic cells were spindle shaped and exhibited mild pleomorphism. The tumor was highly vascularized and its stroma exhibited richly collagenized fibrous connective tissue and myxoid areas (Figure 4). The histopathological findings indicated a mesenchymal neoplasm of uncertain origin.

**Figure 5:**
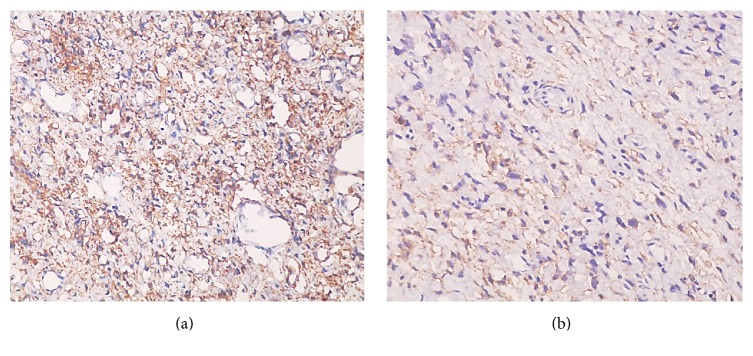
Immunohistochemistry. (a) Positive staining for CD34. (b) Positive staining for CD99.

Immunohistochemical analysis showed positive staining for CD34 and CD99 and negative staining for *α*-SMA, S-100, and bcl-2. Based on the combination of histopathological and immunohistochemical features, the final diagnosis was SFT (Figure 5).

The patient had good postoperative evolution and is currently under clinical follow-up without signs of recurrence after surgery.

## 3. Discussion

First described by Klemperer and Rabin in 1931, SFT is a rare mesenchymal neoplasm of variable clinical behavior [12]. The diagnosis of SFT affecting extrapleural sites is difficult because of the nonspecific clinical and microscopic features of the tumor [3, 8–12].

SFT of the head and neck region is rare. A recent report describing 153 cases of SFT in the head and neck demonstrated that the most frequently involved sites are the buccal mucosa (26.1%), nasal cavity (9.2%), pharyngeal area (7.8%), and tongue (7.2%) [7]. Clinically, these lesions in oral cavity present as a well-circumscribed submucosal mass, asymptomatic, and can often be confused with other lesions.

SFT is rare in the floor of the mouth and usually appears as a slow-growing, painless, well-defined, and mobile swelling (Table 1). Our case is the sixth case described in the literature and differs from the other cases in the fact that the patient reported pain. However, pain symptomatology is a less common finding in intraoral SFTs.

The most common microscopic findings of SFT are a storiform growth pattern, spindle-shaped cells without atypia, alternation between densely cellular and hypocellular areas, and prominent hemangiopericytoma-like branching vascularization [4]. The histopathological findings of the present case are similar to those described in the literature. However, considering that extrapleural SFTs are rare and the histopathological findings are nonspecific, the use of an immunohistochemical panel for confirmation or elucidation of the diagnosis is recommended [4, 7, 13].

SFT exhibits strong immunostaining for CD34. However, CD34 is not specific since it is also a sensitive marker for other neoplasms such as dermatofibrosarcoma and Kaposi sarcoma. Thus, a combination of positive immunostaining for CD34, CD99, and bcl-2, as well as negative staining for muscle, epithelial, and neural markers, is characteristic [3, 9–11]. The most common immunohistochemical profile is positive staining for CD34 and bcl-2 or for CD34, bcl-2, and CD99. Leonardo et al. [14], studying 18 extrapleural SFTs, observed coexpression of CD34 with CD99 or bcl-2 in 100% of cases.

No immunoexpression of bcl-2 was observed in the present case. Studies report immunostaining for bcl-2 in about 80% of SFTs [4, 14]. However, negative immunostaining for bcl-2 does not rule out the diagnosis of SFT and the demonstration of positive staining for CD34 and CD99 is necessary in these cases [10–12], as observed in the present study.

Local excision is the treatment of choice and profuse bleeding during the surgical procedure is common. SFT can develop a more aggressive clinical behavior and its prognosis is based on the presence or absence of histological findings of malignancy such as high cellularity, a mitotic index higher than 4 mitoses per 10 fields at high magnification, presence of necrosis, and cellular pleomorphism [11, 13–16]. The use of adjuvant therapies such as radiotherapy and chemotherapy has been reported in cases in which surgical excision was not possible [6, 7].

Periodical follow-up of patients diagnosed with SFT is indicated because of the variable clinical behavior of the tumor, including recurrences and, in rare cases, distant metastases [4]. The present patient is under follow-up and no signs of recurrence of the neoplastic process were observed 7 months after surgery.

Although uncommon, SFT should be included in the differential diagnosis of lesions in the oral cavity. The combination of clinical-pathological and immunohistochemical features is important to establish the diagnosis of the tumor. Correct treatment and follow-up of the patient by the responsible professional team are important for a favorable prognosis.

## Figures and Tables

**Table 1:** Characteristics of solitary fibrous tumor in the floor of the mouth.

Reference	Year	Age (years)	Sex	Symptoms	Clinical features	Diagnostic hypothesis	Treatment
Ogawa et al. [8]	2003	59	M	Asymptomatic	Well-defined, mobile nodule measuring 3.8 × 3 cm	Ranula/benign tumor of glandular origin	Surgical excision
Shine et al. [9]	2006	35	F	Pain	Well-defined, mobile nodule measuring 3 × 4 cm	NI	Surgical excision
Ayad and Ghannoum [10]	2007	74	F	Asymptomatic	Well-defined, mobile nodule measuring 3 cm	Ranula	Surgical excision
Shi and Wei [11]	2015	39	F	Asymptomatic	Well-defined, mobile nodule measuring 3 × 4 cm	NI	Surgical excision
Carlos et al. [4]	2016	70	F	NI	Well-defined nodule measuring 4 cm	NI	Surgical excision
*Present case*	*2016*	*66*	*F*	*Pain*	*Well-defined, mobile nodule measuring 4 cm.*	*Pleomorphic adenoma*	*Surgical excision*

F: female; M: male; and NI: not informed.
